# Association between Physical Activity Knowledge and Levels of Physical Activity in Chinese Adults with Type 2 Diabetes

**DOI:** 10.1371/journal.pone.0115098

**Published:** 2014-12-10

**Authors:** Stanley Sai-Chuen Hui, Grace Pui-Sze Hui, Yao Jie Xie

**Affiliations:** 1 Department of Sports Science and Physical Education, The Chinese University of Hong Kong, Hong Kong, Hong Kong SAR, China; 2 Department of Medicine and Geriatrics, United Christian Hospital, Hong Kong, Hong Kong SAR, China; University of Westminster, United Kingdom

## Abstract

**Background:**

Physical activity (PA) is an important treatment regimen for diabetes. The purposes of this study were to evaluate people’s knowledge of how exercise influences wellbeing (termed “PA knowledge” or “knowledge of PA” in this paper) and the resulting association with levels of PA in Chinese adults with Type 2 diabetes, and to identify the valuable demographic and lifestyle factors that possibly influence the association between PA knowledge and level of PA.

**Methods:**

Two hundred and fifty-eight adults with Type 2 diabetes completed an interviewer-administered survey at a diabetes clinic in Hong Kong. Data on demographics, lifestyle factors and diabetes-related medical indicators were obtained. A 20-item questionnaire was developed to measure PA-related knowledge (one point scored for each correct answer; aggregate score up to 20 points). level of PA was measured by the International Physical Activity Questionnaire.

**Results:**

The proportions of correct answers to each question ranged from 19.4 to 90.7%. Compared with poorly educated participants, those with university education level and above had PA knowledge scores 1.7 points higher (14.3 *vs.* 12.6, *P<*0.05). Younger, female, and obese participants were more likely to have lower level of PA (all *P<*0.05). After adjustment for age, gender, (BMI) and education level, the odds of having a moderate-to-high level of PA was 19% greater with 1 unit increase in PA knowledge score [95% confidence interval (CI): 1.09–1.29; *P<*0.001], this association was strongest in participants with tertiary education level or above [odds ratio (OR): 1.35; 95% CI: 1.03–1.77; *P<*0.05].

**Conclusions:**

PA knowledge was positively associated with level of PA. Education level significantly influenced the association between PA knowledge and level of PA, leading to the suggestion of vulnerable groups to target for PA improvement in the face of diabetes.

## Introduction

Diabetes mellitus (DM) poses a major threat in the global burden of disease. It caused 5.1 million deaths in 2013 [1]. Worldwide, the total number of people with diabetes is projected to rise from 171 million in 2000 to 592 million in 2035. Approximately 70% of this growth is predicted to occur in the developing world, as is now happening in Asia [1], [2]. In 2013, 9.5% of the Hong Kong population aged 20–79 years had diabetes [1], Hong Kong was one of the 10 regions in the world with the highest prevalence of diabetes among adults [1]. The number of people with diabetes is increasing because of population growth, extended lifespans, urbanization, and the increasing prevalence of obesity and physical inactivity, [1], [2].

Physical activity (PA) is the cornerstone of lifestyle modification aimed at preventing and managing Type 2 diabetes and its related morbidities. PA has been shown to improve glycemic control through increased insulin sensitivity and glucose tolerance [3]. Evidence from randomized, controlled trials has demonstrated that maintenance of modest weight loss through PA and diet reduce the incidence of Type 2 diabetes in high-risk individuals by as much as 40−60% [4]–[6], and are more effective than pharmacological interventions [6]. The risk of mortality among individuals with diabetes is also inversely related to fitness level [7]. Current guidelines firmly recognize the therapeutic strength of exercise intervention [8].

Despite evidence of the benefits of exercise, the majority of people with Type 2 diabetes are physically inactive [9]. In Western countries, for example, data from a national health survey in the U.S.A. found that less than one-third of diabetic adults, who exercised voluntarily, met the recommended levels of PA [10]. Another nationally representative cohort study also showed that people with diabetes were less likely to meet recommendations for PA than those without the disorder [11]. Furthermore, they were more prone to relapse into sedentary behaviour when attempting lifestyle change [12]. In Hong Kong, a recent survey by the Department of Health showed that 23.2% of the general population had a low level of PA [13]; however, few studies reported the levels of PA in adults with diabetes. It is thus important to know the levels of PA and its correlates in Hong Kong’s diabetic adults, as well as to make efforts to boost participation in physical activity.

Previous studies have suggested that many demographic characteristics and lifestyle factors tend to be associated with the participation in PA, such as gender, age, education level, and race [9], [14]. The knowledge of PA, in other words, awareness and understanding of its benefits, may also have an impact on aspects of daily life that are considered to be crucial to participation [15]. A study on Hong Kong Chinese adults indicated that increasing understanding of the effects of PA, particularly knowledge about appropriate exercise prescription, is a positive factor in improving participation levels [16]. However, contradictory findings were found in the literature on the relationship between knowledge of PA and adherence to a suitable regimen [9], [17]–[19]. The effect of knowledge deficits relating to PA need to be further addressed. In short, to effectively promote the adoption and maintenance of PA for diabetes adults, more evidence is needed on the demographics, health, lifestyle, and cognitive factors that influence PA behaviour. The objectives of this study were: (i) to evaluate the PA knowledge and levels of PA in a sample of Hong Kong Chinese adults with Type 2 diabetes, (ii) to examine the association between knowledge and exercise level, and (iii) to identify the valuable demographic and lifestyle factors that possibly influence the association between PA knowledge and level of PA.

## Methods

### Participants

Chinese adults with Type 2 diabetes who attended the diabetes clinic of a local regional hospital from January to July 2010 were enrolled in this study. The exclusion criteria were (i) less than 1 year of diabetic history, (ii) women who were diagnosed with diabetes only during pregnancy and (iii) inadequate understanding of Chinese language. All participants were asked to complete an interview-based questionnaire during their visit to the clinic. For participants who had difficulty understanding or were unable to read the questionnaire, the trained interviewer read and explained it to them. The study protocol was approved by the Research Ethics Committee of the Chinese University of Hong Kong. Written consent was obtained from each participant.

### Measures

#### Physical activity knowledge

The measurement of PA knowledge was created from experts’ consultations (i.e. a psychologist, an endocrinologist, a physiotherapist and a sports science expert), focus group discussion by investigators, and the published research literature [9], [20]. This preparatory work was done to learn more about the psychology, behavioural and environmental barriers to participation in PA among adults with diabetes. We then developed a questionnaire by summarizing ideas from these preparatory works, as well as referring to previous studies by Hui *et al.* [16] and Morrow *et al.* [21]. The questionnaire consisted of 20 items that asked respondents about (i) basic understanding of PA’s benefits (items 1 and 9–11), (ii) health benefits of PA in respect of diabetes (items 2–6), (iii) details of PA for diabetes treatment (items 7 and 8) and (iv) the types of PA conferring health benefits in cases of diabetes (item 12a–item 12i). The choices of response were “Agree”, “Disagree”, and “Don’t know”. The participants scored one point for each correct response and zero for an incorrect or “I don’t know” response. The respondent’s score out of 20 determined his or her degree of understanding of the influences of PA on diabetes-related health. The validity of the questionnaire content was confirmed by the sports science and diabetes management experts. The pilot test of this questionnaire achieved high internal consistency and reliability (Cronbach’s Alpha: 0.86).

#### Physical activity level

International Physical Activity Questionnaire (IPAQ), in its Chinese short-form version [22], was used to measure level of PA. The IPAQ was developed for people aged 15–69 years. The short form measures PA across all domains of leisure time, work, transportation, and household tasks. It asks the respondents to report duration (in minutes) and frequency (days) of walking, moderate-intensity and vigorous-intensity activity, performed for at least 10 minutes per session, during the previous 7 days. The IPAQ short form was considered flexible enough to be used in telephone interviews or in self-administered applications, and adaptable enough to apply across cultures [22]. Standardized pictures were used to depict types or intensities of different physical activities. Reported minutes per week in each category were expressed in metabolic equivalents (METS), resulting in a PA estimate independent of body weight and expressed in MET-minutes/week. The categorical score that refers to MET-minutes/week and the duration and frequency of PA, was then calculated to classify the participants into three levels of PA: low, moderate and high [23].

#### Demographic characteristics, lifestyle factors, health status and diabetes-related indicators

Respondents were asked about their demographic characteristics and lifestyle factors, including age, sex, education level, monthly household income, smoking habit, and marital status. Other chronic diseases in addition to Type 2 diabetes were determined by screening medical records for the medical diagnoses of any of the following disorders: hypertension, hypercholesterolaemia, ischaemic heart disease, and stroke. Duration of diabetes, current mode of treatment, level of glycaemic control (HbA1C), pharmacological treatment for diabetes, weight and height were also determined from medical records. Body mass index (BMI) was then calculated: individuals were considered obese if their BMI was ≥25 kg/m^2^ and were considered as overweight if their BMI was 23–24.9 kg/m^2^ [24]. Glycaemic control was measured using glycosylated haemoglobin (HbA1C) levels, and was reported for the 12 months preceding the interview.

### Statistical Analysis

Descriptive statistics were presented as mean and standard deviation (SD) for continuous variables and percentage for categorical variables. The proportions of false/true/unknown answers for each PA knowledge question were calculated. One-way analysis of variance (ANOVA) was conducted to compare PA knowledge score against different demographic characteristics and lifestyle factors. For variables with more than two categories, *post-hoc* comparisons were used. The demographic characteristics and lifestyle factors, as well as the PA knowledge scores and diabetes-related medical indicators in the three PA levels were compared by Chi-squared test or one-way ANOVA as appropriate. Binary logistic regression analyses were performed to calculate odds ratios (OR) and 95% confidence intervals (CI) for adequate (moderate or high) levels of PA per unit increase in PA knowledge score. The OR was obtained from essentially two comparisons: 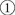 low PA *vs.* (moderate + high) PA; 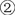 low PA *vs.* moderate PA; 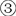 low PA *vs.* high PA. Those demographic characteristics and lifestyle factors that were significantly associated with level of PA in univariate analysis were adjusted in the logistic regression models. To test the moderation of the association between PA knowledge and level of PA by demographic characteristics and lifestyle factors, interaction terms were constructed for those that had significant associations with PA knowledge and level of PA in univariate analysis, and then tested individually in the logistic regression model. Stratified models were subsequently constructed to elucidate the direction of moderation. Statistical analyses were performed in SPSS 19.0 (SPSS Institute): *P<*0.05 was considered to be statistically significant.

## Results

A total of 290 patients were interviewed. After discarding 32 incomplete or duplicated cases, the final data included 258 cases for analysis (S1 File). The samples consisted of 151 men (58.5%) and 107 women (41.5%), with a mean age of 51.6±10.4 years. The mean duration of diabetes was 10.2±7.3 years. The majority of the samples had a BMI ≥23 kg/m^2^, with 19.4% classified as overweight (BMI 23–24.9 kg/m^2^), 34.1% as obese I (BMI 25–29.9 kg/m^2^), and 21.2% as obese II (BMI ≥30 kg/m^2^). Altogether, 62.4% required insulin injection; however, only one-fifth of the participants had an optimal HbA1C level (<7%). Table 1 shows the descriptive statistics of participants by gender groups. Compared with men, women had a lower education level and longer duration of DM, and were less likely to be smokers (all *P<*0.05).

**Table 1:** Descriptive statistic of participants (*n* = 258).

Variables	Men	Women
***n***	151	107
**Age, mean (SD)**	51.3 (10.1)	51.9 (10.8)
**BMI (kg/m^2^), mean (SD)**	26.5 (4.9)	26.7 (5.3)
**BMI categories, ** ***n*** ** (%)**		
Underweight <18.5	4 (2.6)	3 (2.8)
Normal 18.5–22.9	32 (21.2)	22 (20.6)
Overweight 23–24.9	30 (19.9)	20 (18.7)
Obese I 25–29.9	53 (35.1)	35 (32.7)
Obese II ≥30	32 (21.2)	27 (25.2)
**Smoking status, n (%)**		
Non-smoker	76 (50.3)**	95 (88.8)
Ex-smoker	41 (27.2)	4 (3.7)
Current smoker	34 (22.5)	8 (7.5)
**Educational level, ** ***n*** ** (%)**		
Secondary education or below	113 (74.8)*	93 (86.9)
Tertiary education	16 (10.6)	10 (9.3)
University degree or above	22 (14.6)	4 (3.7)
**Duration of DM (years), mean (SD)**	9.4 (7.3)*	11.3 (7.3)
**HbA1C (%), mean (SD)**	8.3 (1.7)	8.4 (1.7)
**DM treatment, ** ***n*** ** (%)**		
OHA	58 (38.4)	33 (30.8)
OHA + insulin	85 (56.3)	67 (62.6)
Other	8 (5.3)	7 (6.6)
**Household income (HK$), ** ***n*** ** (%)**		
<$10 000	58 (39.5)	47 (46.6)
$10 000 – $19 999	47 (32.0)	36 (35.6)
≥$20 000	42 (28.5)	18 (17.8)
**Marital status, ** ***n*** ** (%)**		
Never married	23 (15.2)	22 (20.6)
Currently married and other	128 (84.8)	85 (79.4)
**Hypertension, ** ***n*** ** (%)**	92 (60.9)	72 (67.3)
**Hyperlipidaemia, ** ***n*** ** (%)**	90 (59.6)	58 (54.2)
**Ischaemic heart disease, ** ***n*** ** (%)**	20 (13.2)	9 (8.4)
**Stroke, ** ***n*** ** (%)**	6 (4.0)	6 (5.6)

DM = diabetes mellitus; OHA = oral hypoglycaemic agents.

Comparison of differences between men and women, *indicates *P<*0.05; **indicates *P<*0.001.

The average PA knowledge score was 12.85±3.46 out of a possible 20, which indicated that the participants could correctly answer more than half of the questions. However, the proportions of three choices of response in each question, as shown in Table 2, indicate a few questions that need attention. Only 19.4% of participants knew that patients with Type 2 diabetes should avoid exercising in the evening. More than half of the participants (59.3%) were not aware that weight lifting, being one form of resistance exercise, can provide health benefits for patients with Type 2 diabetes (item 12i). About two-thirds of participants demonstrated limited knowledge on the effects of resistance exercises on diabetes management (items 7 and 8). More than one-third of the participants (37.6%) incorrectly believed that preparing meals was a PA that could provide health benefits (item 12g). Differences in PA knowledge scores according to demographic and lifestyle factors are illustrated in Table 3. Participants with higher education level had higher PA knowledge scores (*P<*0.05). Compared with lowly educated (Secondary education or below) participants, those with university- or higher education levels scored 1.7 points higher in their PA knowledge scores (14.3 *vs.* 12.6; *P<*0.05). No significant differences were observed according to gender, age group, BMI category, smoking status, income and marital status (all *P*>0.05).

**Table 2:** Proportions of different answers in each physical activity knowledge question.

Questions^a^	Answers		
	Agree *n* (%)	Disagree *n* (%)	Don’t know *n* (%)
single session of 30 minutes. (T)	134 (51.9)	84 (32.6)	40 (15.5)
physical activity most days of the week. (T)	201 (77.9)	26 (10.1)	31 (12.0)
provide health benefits. (F)	193 (74.8)	41 (15.9)	24 (9.3)
4. Patients with Type 2 diabetes should be physically active at least 5 days a week. (T)	187 (72.5)	37 (14.3)	34 (13.2)
5. Patients with Type 2 diabetes should avoid exercising in the evening. (T)	50 (19.4)	142 (55.0)	66 (25.6)
6. Regular exercise or being physically active helps to control your diabetes. (T)	234 (90.7)	12 (4.7)	12 (4.7)
7. Patients with Type 2 diabetes should have resistance trainingthat involves all major muscle groups. (T)	90 (34.9)	92 (35.7)	76 (29.5)
8. Resistance training can improve insulin resistance and increase insulin sensitivity. (T)	87 (33.7)	66 (25.6)	105 (40.7)
9. Greater health benefits can be achieved by increasing the amount (duration, frequency, or intensity)of physical activities. (T)	143 (55.4)	82 (31.8)	33 (12.8)
10. Performing physical activities only on weekends is enough to achieve health benefits. (F)	200 (77.5)	35 (13.6)	23 (8.9)
11. Performing vigorous physical activities for 3 hours once aweek is enough to experience health benefits. (F)	204 (79.1)	24 (9.3)	30 (11.6)
12. Which of the following physical activities do you believe will provide health benefits?			
a. aerobics class (T)	198 (76.7)	31 (12.0)	29 (11.2)
b. biking (T)	220 (85.3)	17 (6.6)	21 (8.1)
c. dancing (T)	182 (70.5)	44 (17.1)	32 (12.4)
d. household cleaning (T)	175 (61.8)	60 (23.3)	23 (8/9)
e. jogging/running (T)	218 (84.5)	26 (10.1)	14 (5.4)
f. playing a musical instrument (F)	148 (57.4)	63 (24.4)	47 (18.2)
g. preparing meals (F)	124 (48.1)	97 (37.6)	37 (14.3)
h. swimming (T)	222 (86.0)	19 (7.4)	17 (6.6)
i. weightlifting (T)	105 (40.7)	115 (44.6)	38 (14.7)

a. T for true; F for false.

**Table 3:** Physical activity knowledge score according to different demographic and lifestyle factors.

	*n*	Physical activity knowledge score, mean (SD)	*P-value* a
**Gender**			0.964
Female	107	12.8 (3.7)	
Male	151	12.9 (3.3)	
**Age group**			0.458
≤40	37	12.9 (2.8)	
41–50	77	13.2 (2.8)	
51–60	91	12.9 (3.8)	
≥61	53	12.2 (4.1)	
**BMI, kg/m^2^**			0.264
Under/normal weight <23	61	12.3 (4.0)	
Overweight 23–24.9	50	13.4 (2.4)	
Obese ≥25	147	12.9 (3.5)	
**Smoking**			0.815
Non-smoker	171	12.8 (3.1)	
Ex-smoker	45	13.1 (3.0)	
Current smoker	42	12.9 (3.1)	
**Educational level**			0.023
Secondary education or below	206	12.6 (3.6)*	
Tertiary education	26	13.6 (2.7)	
University degree or above	26	14.3 (2.2)*	
**Household income (HK$)**			0.689
<$10 000	105	12.8 (3.6)	
$10 000–19 999	83	12.9 (3.0)	
≥$20 000	60	13.1 (3.4)	
**Marital status**			0.827
Never married	45	13.0 (3.5)	
Currently married and other	213	12.8 (3.5)	

a *P-*values were generated by one-way ANOVA.

*post hoc comparison was used; difference between two groups was significant (*P<*0.05).

Table 4 shows that the majority of the participants (70%) reported moderate and high PA: in other words, 30% did not engage in sufficient PA. Significant differences were found in levels of PA between participants in different gender, age, and BMI categories. More men than women reported high levels of PA (18.5% in males *vs.* 9.3% in females; *P<*0.05). Older people tended to participate more in PA (*P<*0.05). Those with low levels of PA were more likely to be obese (*P<*0.05). Only two highly educated individuals (0.7% of the total) reported high PA. Of highly educated participants, 34.6% had low PA levels, whereas this proportion was 26.7% among poorly educated participants (Table 4). No significant difference was found in diabetes-related medical indicators between the three leves of PA groups (data not shown). A significant difference in PA knowledge score was found: participants reporting high levels of PA had higher PA knowledge scores than those reporting moderate and low levels (*P<*0.01) (Table 4).

**Table 4:** Physical activity level according to different demographic and lifestyle factors.

Variables	Physical activity level			*P-*value
	Low	Moderate	High	
**Gender, ** ***n*** ** (%)**				0.026
Female	27 (36.0)	70 (48.3)	10 (26.3)	
Male	48 (64.0)	75 (51.7)	28 (73.7)	
**Age, mean (SD)**	49.3 (10.4)*	53.0 (10.1)*	51.9 (10.8)	0.035
**BMI (kg/m^2^), ** ***n*** ** (%)**				0.023
Under/normal weight <23	19 (25.3)	33 (22.8)	9 (23.7)	
Overweight 23–24.9	5 (6.7)	35 (24.1)	10 (26.3)	
Obese ≥25	51 (68.0)	77 (53.1)	19 (50.0)	
**Smoking, ** ***n*** ** (%)**				0.280
Non-smoker	52 (69.3)	96 (66.2)	23 (60.5)	
Ex-smoker	8 (10.7)	27 (18.6)	10 (26.3)	
Current smoker	15 (20.0)	22 (15.2)	5 (13.2)	
**Educational level, ** ***n*** ** (%)**				0.058
Secondary education or below	55 (73.3)	122 (84.1)	29 (76.3)	
Tertiary education	11 (14.7)	8 (5.5)	7 (18.4)	
University degree or above	9 (12.0)	15 (10.3)	2 (5.3)	
**Household income (HK$), ** ***n*** ** (%)**				0.506
< $10000	30 (41.7)	62 (44.6)	13 (35.2)	
$10000 – $19999	22 (30.6)	49 (35.3)	12 (32.4)	
≥ $ 20000	20 (27.8)	28 (20.1)	12 (32.4)	
**Marital status, ** ***n*** ** (%)**				0.198
Never married	18 (24.0)	22 (15.2)	5 (13.2)	
Currently married and other	57 (76.0)	123 (84.8)	33 (86.8)	
**Physical activity knowledge score, mean (SD)**	11.6 (4.3)**	13.4 (3.0)**	13.5 (2.7)**	0.001

*post hoc comparison was used, group difference (low *vs.* moderate) was significant at *P<*0.05 level.

**post hoc comparisons were used, group differences (low *vs.* moderate; low *vs.* high) were significant at *P<*0.01 level.

The logistic regression analysis results are shown in Table 5. The crude odds of exhibiting an adequate (moderate or high) level of PA were 16% greater with a 1 point increase in PA knowledge score (OR: 1.16; 95% CI: 1.07–1.25; *P<*0.001). After adjusting for gender, age, BMI and education level, the OR increased to 1.19 (95% CI: 1.09–1.29; *P<*0.001). The subgroup analysis also showed significant positive associations between PA knowledge and level of PA: the ORs were 1.19 (95% CI: 1.0–1.28; *P<*0.001) and 1.21 (95% CI: 1.06, 1.39; *P<*0.01) for a moderate PA level and high PA level, respectively. No significant interaction was observed between PA knowledge and age, gender, BMI on level of PA (all *P*>0.05; data not shown). In the univariate analysis, only education level showed significant associations with PA knowledge. The stratified analysis was then conducted by categorizing participants into two education levels (‘tertiary education’ and ‘university degree or above’ were combined into one category, owing to the limited number of samples). As shown in Table 5, we observed the strongest association between PA knowledge and level of PA in participants with tertiary education level or above (OR: 1.35, 95% CI: 1.03–1.77; *P<*0.05).

**Table 5:** Odds ratios (ORs) for level of PA per unit increase in PA knowledge score by logistic regression analysis.

Model	N	OR (95% CI)	*P*-value
**PA knowledge with low/(moderate + high) level of PA** a	258		
Model 1^e^		1.16 (1.07, 1.25)	<0.001
Model 2^f^		1.17 (1.08, 1.27)	<0.001
Model 3^g^		1.18 (1.08, 1.28)	<0.001
Model 4^h^		1.19 (1.09, 1.29)	<0.001
**PA knowledge with low/moderate level of PA** b	220		
Model 1^e^		1.15 (1.06, 1.25)	0.001
Model 2^f^		1.17 (1.07, 1.27)	<0.001
Model 3^g^		1.17 (1.07, 1.27)	<0.001
Model 4^h^		1.19 (1.09, 1.28)	<0.001
**PA knowledge with low/high level of PA** c	113		
Model 1^e^		1.17 (1.03, 1.33)	0.019
Model 2^f^		1.17 (1.03, 1.33)	0.016
Model 3^g^		1.19 (1.03, 1.36)	0.010
Model 4^h^		1.21 (1.06, 1.39)	0.006
**Stratified model by education level** d			
Model 5^i^	206	1.18 (1.08, 1.30)	<0.001
Model 6^j^	52	1.35 (1.03, 1.77)	0.030

a level of PA was classified as two levels: low and moderate + high.

b level of PA was classified as two levels: low and moderate.

c level of PA was classified as two levels: low and high.

d Stratified model by education level was conducted, logistic regression analyses were conducted respectively among participants in two education levels: secondary education or below, tertiary education or above.

e Crude OR for level of PA per unit increase in PA knowledge score was calculated.

f Model was adjusted for gender and age.

g Model was adjusted for gender, age and BMI.

h Model was adjusted for gender, age, BMI and education level.

i Analysis was conducted in participants with secondary education level or below. Model was adjusted for gender, age and BMI.

j Analysis was conducted in participants with tertiary education level or above. Model was adjusted for gender, age and BMI.

## Discussion

In this study, effort was made to develop a questionnaire for evaluating the factual knowledge of PA among Chinese adults with Type 2 diabetes, particularly as relating to the benefits or risks conferred on their health through active participation. A significant positive association between PA knowledge and level of PA was found. Those more knowledgeable about appropriate exercise prescription exhibited higher levels of PA than those who were short of relevant knowledge. This finding is contrary to those previous studies that showed a lack of a direct association between PA knowledge and reported weekly duration of PA [9], [25]. One of the previous studies pointed out that the deficient assessment of knowledge might be the reason for observing a poor relationship between knowledge and behaviour [9]. Thus a definite and targeted system is needed for measurement for PA knowledge. We designed a 20-item diabetes-specific questionnaire to assess the patients’ awareness and understanding of PA’s benefits for DM. We suggest that it is a concise and effective instrument to evaluate the PA knowledge among diabetic patients, which could help us to better understand their perceptions of their PA and its relationship with DM. To our knowledge, this is the first study that developed a PA knowledge assessment questionnaire specifically for adults with Type 2 diabetes. The results showed that participants lacked knowledge of how to exercise to achieve health benefits, especially the benefits that may be derived from resistance training. Also, they had little knowledge that enable them to identify specific PA behaviours associated with improved health. Therefore, more studies and analyses must be made of the types, frequencies, intensities, and durations of appropriate forms of PA when investigating the benefits of exercise to diabetic patients.

Education level was found to positively associate with PA knowledge in this study. This supports the view of Dishman [18] and Sallis *et al.* [19], that increasing PA knowledge through education is an effective method of promoting PA. Also, participants with tertiary or above education level demonstrated a 17% higher likelihood of doing sufficient (moderate or high-level) PA than those with lower education levels (OR: 1.35 *vs.* 1.18), from a one-point increase in PA knowledge score (i.e. one more correct answer out of the twenty questions). This implies that well-educated people may have more willpower to change their behaviour when they are given appropriate, beneficial knowledge. In other words, a PA education program among well-educated people may achieve better efficiency in promoting their PA. This finding also reminds us that programmes for education in–and promotion of–PA should be strengthened for diabetic patients who have low levels of education.

In our study, older participants reported higher levels of PA. This result corresponds with the findings in non-diabetic Chinese individuals [16], [26], revealing that middle-aged Chinese had the lowest rates of sports participation (43%), showing an upward trend beginning at age 45, and rising to 58% for those aged 65 or above. These results are clearly different from the findings reported in many western countries [27], [28]. Thomas *et al.* showed that inactivity in older patients was associated with lack of self-motivation, feelings of tiredness, and distraction by good television programs. All these factors were important to the younger patients and lack of time, fear of worsening diabetes, poor weather, and feeling depressed were also causes associated with inactivity in the younger group [28]. Younger subjects may also perceive their health as being good [29]. On the other hand, older people are more concerned about their health [30]. Hence, efforts have to be made to encourage sedentary young adults with Type 2 diabetes to increase their PA. Educating individuals with Type 2 diabetes about the benefits of PA on diabetes control at a younger age is especially important. The present study has also shown that more male participants than females were in the ‘high level of PA’ group. A previous study has shown that men are more likely than women to participate in leisure time and occupational activities [31], which explains why more male participants were in the ‘high PA’ group, whereas their female counterparts–consistent with their traditional gender roles–would apply themselves to housework and shopping [32], which are categorized as ‘moderate PA’.

There are several limitations in our study. First, the self-reported PA behaviour may lead to a certain extent of misclassification [33]. With self-reported metrics, the possibility exists of deliberate or unconscious misinformation. Nevertheless, IPAQ has been found to be a valid tool for PA measurement in Type 2 diabetes patients, without recourse to more sophisticated methods [34]. Second, although the PA knowledge questionnaire developed in this study provides a general assessment of exercise knowledge for diabetic patients, further investigation on its construct validity is needed, and more comprehensive and precise measurements are suggested. Besides knowledge, other variables associated with PA behaviour, including self-motivation, outcome expectation, and social support [35], should also be considered, and appropriate strategies incorporated. Finally, the relatively small sample size, as well as the convenience sampling method used in our study, limited the general applicability of the study.

Despite these limitations, this study is one of the first to develop a diabetes-specific questionnaire to assess the PA knowledge and estimate its association with level of PA in Chinese adults with Type 2 diabetes. The study provides a unique questionnaire template that can be further developed in future study to comprehensively assess diabetic patents’ perceptions, attitudes, and intentions on improving their behaviour in respect of PA. The study adds reliable evidence to the literature, in that PA knowledge was positively associated with level of PA in Chinese adults with Type 2 diabetes, which indicates that improving patients’ perceptions on PA is a key process in improving their levels of participation in PA. We suggest that future studies are needed, into devising tailor-made health behaviour interventions to improve PA levels, based on patients’ different PA knowledge status. In summary, the findings of the present study set out a reference for future research into PA knowledge and identifying ways of increasing levels of PA in adults with diabetes.

### Implications and relevance for diabetes educators

Given the importance of PA in the management of diabetes, these results have important implications for healthcare provider, in terms of interventions that help increase levels of PA in similar populations. Healthcare providers may need to spend more time educating people about the health benefits of PA and in encouraging them to exercise. The positive effects of PA on diabetes management should be the key message, to help healthcare providers develop appropriate exercise programs based on individual capabilities, physical limitations, and personal interests. Education level significantly influenced the association between PA knowledge and level of PA in our study, suggesting that diversified and specific PA promotion and education programs should be developed and strengthened in vulnerable education level groups.

## Supporting Information

S1 File Data of the 258 subjects who participated in the survey.
(SAV)
