# Correction: Association between Physical Activity Knowledge and Levels of Physical Activity in Chinese Adults with Type 2 Diabetes

**DOI:** 10.1371/journal.pone.0124701

**Published:** 2015-04-07

**Authors:** The PLOS ONE Staff

There are errors in Questions 1–3 in Table 2, “Proportions of different answers in each physical activity knowledge question.” Please see the corrected Table 2 here. The publisher apologizes for these errors.

**Table 2:** Proportions of different answers in each physical activity knowledge question.

Questions^a^	Answers		
	Agree *n* (%)	Disagree *n* (%)	Don’t know *n* (%)
1. Ten minutes of physical activity three times per day provide the same health benefits as a single session of 30 minutes. (T)	134 (51.9)	84 (32.6)	40 (15.5)
2. Patients with Type 2 diabetes should get 30 minutes of moderate physical activity most days of the week. (T)	201 (77.9)	26 (10.1)	31 (12.0)
3. Vigorous levels of physical activity are necessary to provide health benefits. (F)	193 (74.8)	41 (15.9)	24 (9.3)
4. Patients with Type 2 diabetes should be physically active at least 5 days a week. (T)	187 (72.5)	37 (14.3)	34 (13.2)
5. Patients with Type 2 diabetes should avoid exercising in the evening. (T)	50 (19.4)	142 (55.0)	66 (25.6)
6. Regular exercise or being physically active helps to control your diabetes. (T)	234 (90.7)	12 (4.7)	12 (4.7)
7. Patients with Type 2 diabetes should have resistance training that involves all major muscle groups. (T)	90 (34.9)	92 (35.7)	76 (29.5)
8. Resistance training can improve insulin resistance and increase insulin sensitivity. (T)	87 (33.7)	66 (25.6)	105 (40.7)
9. Greater health benefits can be achieved by increasing the amount (duration, frequency, or intensity) of physical activities. (T)	143 (55.4)	82 (31.8)	33 (12.8)
10. Performing physical activities only on weekends is enough to achieve health benefits. (F)	200 (77.5)	35 (13.6)	23 (8.9)
11. Performing vigorous physical activities for 3 hours once a week is enough to experience health benefits. (F)	204 (79.1)	24 (9.3)	30 (11.6)
12. Which of the following physical activities do you believe will provide health benefits?			
a. aerobics class (T)	198 (76.7)	31 (12.0)	29 (11.2)
b. biking (T)	220 (85.3)	17 (6.6)	21 (8.1)
c. dancing (T)	182 (70.5)	44 (17.1)	32 (12.4)
d. household cleaning (T)	175 (61.8)	60 (23.3)	23 (8/9)
e. jogging/running (T)	218 (84.5)	26 (10.1)	14 (5.4)
f. playing a musical instrument (F)	148 (57.4)	63 (24.4)	47 (18.2)
g. preparing meals (F)	124 (48.1)	97 (37.6)	37 (14.3)
h. swimming (T)	222 (86.0)	19 (7.4)	17 (6.6)
i. weightlifting (T)	105 (40.7)	115 (44.6)	38 (14.7)

^a^. T for true; F for false
